# Addressing the Medicolegal Aspects of Temporal Lobe Epilepsy: A Literature Review

**DOI:** 10.7759/cureus.76310

**Published:** 2024-12-24

**Authors:** Noé López-Amador, Rosario Castro-Morales, Patricia Denis-Rodríguez, Octavio Carvajal-Zarrabal

**Affiliations:** 1 Department of Clinical and Forensic Neuroscience, University of Veracruz, Boca del Río, MEX; 2 Master’s Degree Program in Forensic Medicine (National Postgraduate System-National Council for Humanities, Sciences, and Technologies), University of Veracruz, Boca del Río, MEX

**Keywords:** criminal behaviour, focal seizures, legal responsibility, sudden death, temporal lobe epilepsy

## Abstract

Temporal lobe epilepsy (TLE) represents a prevalent form of focal epilepsy that often requires surgical intervention and can be resistant to antiseizure medications. Its epidemiology varies across regions due to diagnostic challenges and underestimation of individual neurological traits. Despite these complexities, TLE accounts for a significant proportion of total epilepsies worldwide. Early discussions on TLE's forensic implications date back to the mid-20th century, highlighting its unique behavioral and mental disturbances during seizures. The International League Against Epilepsy categorizes seizures based on type and onset, aiding in diagnosis and treatment planning. Neuroimaging techniques play a crucial role in identifying the epileptogenic focus within the temporal lobe. TLE's etiology involves various risk factors, including genetic predisposition and neurological insults. Clinically, TLE presents diverse signs and symptoms across different phases. This literature review explores the forensic implications of TLE, including legal responsibility, sudden death, and its association with criminal behavior. It examines the challenges in diagnosing TLE in court and discusses the complex interplay between TLE, psychosis, and substance use in determining legal liability. Furthermore, it addresses the risk of sudden unexpected death in epilepsy (SUDEP) and sudden unexplained death in childhood (SUDC) associated with TLE. Finally, the review underscores the need for further research to comprehensively understand TLE's forensic and medicolegal implications, considering its intricate neurobiological underpinnings and clinical manifestations.

## Introduction and background

Temporal lobe epilepsy (TLE), the most prevalent form of focal epilepsy, often necessitates surgical intervention and frequently exhibits resistance to antiepileptic medications. The epidemiology of TLE is approximated, with variations observed across regions and populations, primarily due to diagnostic complexities and the underestimation of specific individual neurological traits. However, TLE accounts for 30-40% of total epilepsies, and epilepsy in general affects at least 1% of the global population [1].

The early discussions concerning TLE in relation to forensic implications have been documented in scientific databases such as PubMed, along with courtroom data reported by Meyer in 1957 [2]. During that period, forensic psychiatrists and neurologists also denoted this condition as "psychomotor" or "psychic" epilepsy. These specialized terms mirrored the distinctive features of TLE seizures, frequently characterized by a blend of behavioral and mental disturbances [2,3].

The classification of the International League Against Epilepsy (2017) categorizes seizures based on their type and onset [4]. This classification distinguishes between focal seizures, which involve localized discharges in the cortex, and generalized seizures, which affect the entire cortex. Generalized seizures are further subdivided into various types. Tonic seizures involve rigid body postures, while clonic seizures are characterized by rhythmic muscle contractions. Tonic-clonic seizures exhibit a combination of both tonic and clonic features, whereas myoclonic seizures result in brief jerking movements, and atonic seizures lead to sudden loss of muscle tone. The classification also encompasses absence seizures, marked by brief lapses in consciousness. Seizure onset is categorized as primary, representing the initial clinical manifestation, or secondary, arising from the spread of a primary seizure. Conversely, simple focal seizures are characterized by preserved consciousness, even if they may include hallucinations or feelings of fear [4].

The terms "temporolimbic" and "limbic" epilepsies, previously utilized by Schomer et al. to describe focal seizures originating in the temporal lobe or limbic system, are now considered a subcategory of TLE based on the epileptogenic focus [5]. The determination of a seizure's neuroanatomical origin relies on a comprehensive analysis of seizure semiology, encompassing a detailed history and video-EEG. Various neuroimaging techniques, such as simple and functional magnetic resonance imaging, fluorodeoxyglucose-positron emission tomography, magnetoencephalography, and invasive intracranial EEG recording, are employed to accurately identify the site of seizure onset. This is particularly relevant for temporal "plus" epilepsy, where the epileptogenic zone extends to regions adjacent to the temporal lobe, also known as temporolimbic epilepsy. Presently, the presence of hippocampal sclerosis in mesial TLE is acknowledged as limbic epilepsy, a clinically relevant subcategory of TLE [6].

Despite the diagnostic challenges posed by TLE, deep EEG recording remains a crucial tool in its diagnosis. This method allows for the identification of seizure onset zones and the mapping of functional brain regions, which are essential for planning surgical interventions. TLE was categorized based on the location of the epileptogenic focus within the temporal lobe. The complex neuronal networks involved in temporal lobe seizures have led to the division of this condition into five subtypes: mesial, temporopolar, mesiolateral, lateral, and temporal "plus". Mesial TLE, often associated with hippocampal sclerosis, is the most common subtype. Temporopolar TLE is linked to the polar region, while mesiolateral and lateral TLE is associated with the lateral temporal cortex. Temporal "plus" epilepsy is a condition where the primary epileptogenic zone is in the temporal lobe and extends to nearby regions. Understanding this classification is crucial for comprehending the varied presentations of TLE and formulating appropriate treatment strategies [4].

Temporal lobe epilepsy (TLE) is multifactorial in origin, with potential etiologies including obstetric trauma, febrile seizures, intracranial infections, and traumatic brain injury. Genetic factors also play an important role, as evidenced by several heritable forms. For example, autosomal dominant lateral temporal lobe epilepsy (ADLTE) is associated with mutations in the LGI1 gene, whereas familial mesial temporal lobe epilepsy (FMTLE) encompasses various subtypes (e.g., hippocampal sclerosis, febrile seizures) but has not yet been conclusively linked to a single gene. Another example is familial partial epilepsy with variable foci (FPEVF), a rare autosomal dominant syndrome mapped to loci on chromosomes 2q and 22q12 [7,8]. These hereditary forms of TLE can often be identified in early disease stages.

Clinically, TLE presents with a diverse array of signs and symptoms across three phases: (a) ictal, (b) interictal, and (c) postictal. These manifestations can encompass motor, sensory, autonomic, experiential, emotional, affective, and cognitive issues. Complex psychiatric phenomena like psychosis and depression may be observed [5].

We aimed to conduct a narrative review of the literature, focusing on significant forensic research related to TLE. The review will explore various forensic implications of TLE, including legal responsibility, sudden death, and other related conditions. A Boolean search was performed using MeSH terms: ("temporal lobe epilepsy" OR "temporal epilepsy" OR "temporolimbic epilepsy" OR "limbic epilepsy" OR "psychomotor epilepsy" OR "psychic epilepsy") AND ("forensic" OR "medicolegal" OR "legal"). The search covered three databases: PubMed/MEDLINE (R=28), Web of Science Core Collection (R=20), and Google Scholar (R=40), and included original articles published at any time that is relevant to the forensic aspects of TLE. By examining the available research from historical, clinical, and expert perspectives, this review aims to provide an updated understanding of TLE in the forensic context. This article was previously posted to the Qeios preprint server on April 3, 2024 [9].

## Review

Temporal lobe epilepsy and its association with criminal behavior

From the mid-20th century onward, numerous studies have established a connection between epilepsy and crime, particularly violent crime [10-12]. Interestingly, classical criminologists like Lombroso proposed a typology of "epileptic criminals," characterizing them as inherently violent, and many of these hypotheses seem to be based on anecdotal case reports [13]. However, comprehensive research by Granieri and Fazio effectively criticizes Lombroso’s theories, while recognizing the potential value of his observations when re-evaluated with advancements in diagnostic and research technology [14].

Effectively, temporal lobe epilepsy is recognized as being associated with certain behavioral conditions. For instance, the practice of exhibitionism [15], psychosis [16], hyposexuality or sexual dysfunction [17,18], and arson [19,20] have been reported in relation to this neurological condition. However, it is crucial to emphasize that an association does not necessarily imply causation. Despite this caveat, the existing literature suggests that behaviors like exhibitionism or arson may occur during the ictal phase of TLE, and psychotic episodes during the postictal phase, as all the reported cases describe symptoms that are clinically consistent with TLE.

The criminological landscape becomes increasingly complex when considering the proposal by Marinacci and Von Hagen that alcohol consumption is strongly associated with temporal lobe dysfunction [21]. This complexity is further compounded when Rosberg and Viukari discuss the relationship between epilepsy, crime, and mental disorders [22]. Facts presented in this order of ideas usually drive discussions of the most controversial positions [21-23].

Considering individual cases where the causal link is clearly established, it appears that the relationship between epilepsy and violence is entirely objective and direct, as illustrated in a case report by Pandya et al. [24]. Following surgical resection, the symptoms of violence that led to the commission of homicide were completely resolved in the patient. This report also documents at least 50 similar cases found in the literature at that time. However, no author has been more adventurous in characterizing the abnormal limbic response for aggression, as a possible link between TLE and violent behavior, than Pontius [25-27]. This author dedicated a lifetime of efforts to study and characterize what is called the "limbic psychotic trigger reaction," a form of non-convulsive behavioral seizures, by clinically examining several homicide cases where the offender was supposed to be under the effects of kindling in the limbic system or temporal lobe. Kindling is a progressive ictal phenomenon initiating from small focal seizures with the potential to grow into a generalized seizure, akin to the ignition of firewood. As Pontius explains, this kindling or non-convulsive reaction produces an imbalance between the temporal lobe, limbic system, and frontal lobe, which translates into the complete loss of control of the patient, triggering an aggressive response without rational motivation [25-27]. Alcohol consumption and drugs can elicit this kindling, adding more complexity to the formula. For this reason, this phenomenon is still a matter of debate among expert neuropsychiatrists, and most of them are cautious about considering it as a proven fact in their expert opinions.

A strong opposition by several authors was raised after the presentation of these hypotheses [28-30], and debating this topic is still a matter of contention today [31]. Multiple pieces of evidence have been presented in observational studies [11,32,33], case series [34,35], and case reports [12,19,36-42]. However, this evidence is not yet strong enough, and opinions remain controversial, as there is a non-unanimous position about TLE as the primary cause of violent or criminal behavior. Most scholars are influenced by the idea of the discriminative use of this notion and the unjustified use of insanity allegations in the courtroom [43].

Indeed, while not all individuals with epilepsy engage in criminal activities, some may exhibit criminal behavior under specific circumstances, including violent acts. An international panel of neurologists has established five criteria to ascertain whether violent and aggressive behavior during a crime could be attributed to an epileptic seizure: (1) Diagnoses of epilepsy must be formulated by at least one neurologist with specific competence in epilepsy; (2) The presence of epileptic automatisms should be documented by clinical history, by a closed-circuit video recording, and electroencephalographic biotelemetry; (3) The presence of aggression during the course of epileptic automatisms should be verified through a video EEG recording, which allows a simultaneous recording of the epileptiform patterns; (4) The violent or aggressive action should be characteristic of the patient’s habitual crises, as should emerge from his clinical history; and (5) The clinical judgment should be drafted by a neurologist, who certifies if it is possible that the aggressive action, the presumptuous crime, is part of an epileptic seizure [43].

The latter criterion seeks to preserve the integrity of expert witnesses in cases involving a purported crime committed under the influence of a seizure, a condition that is exceedingly difficult to ascertain. The five criteria mentioned earlier do not facilitate this process. Thus, establishing a connection between TLE and violence proves challenging both in academic discourse and within the courtroom. In a subsequent section dedicated to this topic, we will endeavor to delve deeper into this discussion.

Temporal lobe epilepsy and the risk of sudden unexpected death in epilepsy

Before delving into the debate surrounding the forensic plausibility of arguing that a crime was committed under the genuine influence of a seizure, it is essential to first explore another significant association of TLE within the forensic practice: its relationship with thesudden unexpected death in epilepsy (SUDEP) syndrome. This syndrome predominantly affects individuals aged 20-45 years, with generalized tonic-clonic seizures posing the greatest risk. Most cases occur during sleep following such seizures, often with victims found in a prone position. SUDEP excludes other forms of seizure-related sudden death, progressing with postictal apnea and bradycardia leading to asystole. Central to SUDEP pathology is brainstem dysfunction, potentially indicated by postictal generalized EEG suppression, alongside factors such as serotonin and adenosine signaling dysfunction, and genetic disorders affecting cardiac conduction and neuronal excitability. Enhanced patient education on preventing tonic-clonic seizures is crucial [44].

Sudden unexplained death in childhood (SUDC), a phenomenon less familiar than sudden infant death syndrome (SIDS), presents as a rare and enigmatic occurrence in children beyond infancy. Recent research suggests a potential connection between SUDC and SUDEP, supported by findings of febrile seizure histories and genetic factors, such as variants in epilepsy-associated genes like SCN1A. Despite these advancements, the precise mechanisms underlying SUDC often discovered posthumously due to the unexpected and unobserved nature of the terminal events, remain speculative. These insights suggest a transition from considering SUDC purely unexplained to one where genetic predisposition and epilepsy-like mechanisms play a role, even in the absence of a documented history of epilepsy [45].

Shorvon and Tomson asserted that the frequency of tonic-clonic seizures correlates with the risk of SUDEP, estimating a twenty-fold increase in the overall risk of sudden death among epileptics compared to the non-epileptic population [46]. Recent investigations into the pathophysiology of SUDEP suggest that autonomic control breakdown during the ictal and postictal phases may underlie the fatal mechanism in the brainstem. An observational study by Mueller et al. linked TLE with volume loss in the dorsal mesencephalon, indicating more extensive brainstem damage and evidence of network breakdown in TLE-SUDEP cases [47]. This underscores the significance of undiagnosed TLE in the genesis of SUDEP. Additionally, Kon et al. identified a potential association between hippocampal abnormalities and epileptic seizures in cases of SUDC through a retrospective cross-sectional study spanning 16 years and involving data from 48 SUDC, 18 SUDEP, and 358 SIDS cases [48].

Furthermore, Kinney et al. delineated the histopathological basis of sudden unexplained death in infants, observing focal granule cell bilamination in 41.2% of cases, starkly contrasting with the 7.7% prevalence in the control group (p < 0.001) [49]. These anomalies co-occurred with other dentate developmental irregularities, implying compromised neuronal proliferation, migration, and/or survival. The authors postulate that in a notable subset of infants experiencing sudden unexplained death, these dentate lesions may signify a developmental susceptibility predisposing them to autonomic/respiratory instability or autonomic seizures, ultimately culminating in sleep-related death when subjected to homeostatic stressors. Notably, these lesions are detectable in microscopic sections prepared using contemporary forensic techniques. Despite such evidence of neural structure abnormalities, some researchers note the absence of autopsy findings distinguishing SUDEP from non-SUDEP deaths [49].

However, despite the anatomical evidence of structural changes observed in SUDEP, we are far from knowing with certainty the underlying cause of this syndrome and other related conditions, such as SUDC. Forensic diagnosis must be cautious, ruling out the presence of other pathologies or intoxication by drugs or poison.

Evaluating temporal lobe epilepsy in court: insanity defense considerations

The most contentious issue surrounding TLE pertains to its utilization as an insanity defense in courtrooms. Determining legal responsibility is a crucial and serious discussion that must be clarified before a jury. When an individual commits a crime while conscious, the legal implications of their actions can vary depending on whether the crime was committed intentionally or due to "accident or disease." This forms the basis of the argument for an insanity defense, which is classically pronounced in the M'Naghten rules: (1) Presumption of sanity and burden of proof, (2) Disease of the mind (defect of reason), (3) Nature and quality of the act, (4) Knowledge that the act was wrong. Even when these rules were first applied in 1843, in the case of Daniel M'Naghten, charged with the homicide of Edward Drummond, erroneously after confusing him with the real target, Prime Minister Robert Peel, at that historical moment, the public, the media, and even Queen Victoria called for a revision of this court decision. The case of M'Naghten is not the best suited to justify the rules that bear his name as an identifier of insanity defense [50].

Focal seizures in epilepsy can manifest as either simple, where the individual remains conscious, or complex, where they may lose consciousness. However, the mental state during TLE is not consistently uniform. Sometimes, patients may recall their actions during a seizure, while at other times, they may not. This inconsistency presents a significant challenge, if not an impossibility, for expert witnesses to determine in court, particularly in cases where there is no documented history of epilepsy or where the commission of a crime during the seizure phases is not captured in video EEG recordings. Cases reported during the interictal phase are even more challenging to assess in a courtroom setting [51].

The insanity defense also encompasses the alteration of consciousness that may occur during a psychotic episode. However, if this condition is induced by alcohol or drug consumption, it may not necessarily exempt an individual from legal liability. TLE or temporal dysfunction can be triggered by alcohol or drugs, as well as the psychotic episode [22,25]. Therefore, being under the influence of voluntary intoxication does not automatically absolve someone of legal responsibility. In this context, the limbic psychotic trigger reaction becomes notably relevant as an argument for psychotic behavior emerging from a postictal phase of TLE or even from the kindling mechanism described by its author [25]. It is essential to recognize that psychotic is not synonymous with psychopath, and not every patient experiencing a psychotic event will be a candidate to commit a crime or exhibit violent behavior.

Another essential aspect for the expert witness to determine is the level of danger associated with an offender who committed a crime under the influence of a TLE seizure, as well as whether the patient can be treated and cured, or if there is a high or low likelihood of recurrence. This responsibility can be overwhelming for the expert witness, and ethical and human rights issues may arise, complicating the balance between the expert's testimony in court and the rights of the patient who is being prosecuted for a crime committed under an involuntary condition that impairs their rational, logical, and moral thinking or memory. Moreover, over all these concerns are the rights of the victim. For instance, restrictions on driver's licenses for epileptics are often implemented as a safety measure in several jurisdictions [52].

The evidence supporting a diagnosis of TLE in court proceedings can come from various sources, including clinical assessments, advanced functional imaging, video-EEG recordings, genetics, or even histopathological examinations [41]. However, the high level of expertise required by the expert witness to interpret these clinical findings can pose a limitation during the legal proceedings. Not all courtrooms or legal authorities rely on the opinion of authentic forensic neuropsychiatrists under their jurisdiction. Sometimes, the responsibility to determine these scientific issues of a crime relies on experts with partial or incomplete clinical and forensic formations to study these cases. Especially in developing societies, this issue may be more pronounced, as resources and specialized expertise in forensic neuropsychiatry may be limited [39]. It must be considered that TLE could coexist with other mental illnesses, and depending on the components, this can severely complicate outcomes in terms of diagnosis and treatment, and of course, forensic implications [53].

## Conclusions

We have presented the most relevant literature evidence on the forensic aspects involving TLE from historical, clinical, and expert perspectives. There is still much work for future research to accomplish, as TLE is an interesting and complex pathology with behavioral, social, and health repercussions. TLE could be underdiagnosed, and the diagnosis must be suggested as suspicious when irrational behavior occurs in a person under certain stressors, recognizing that they were compelled almost involuntarily to misbehave. The five expert criteria for determining that a crime was committed because of a TLE seizure must always be met to ensure an objective and transparent expert witness testimony. TLE could manifest as a single diagnosis or as the focal seizure that ignites a generalized one. Sometimes TLE manifestations are exclusively behavioral, and prodromal behavior is very subtle. The medical examiner should be aware of this diagnosis in cases of SUDEP or even in SUDC. Further research is still necessary to comprehensively study this condition and its forensic and medicolegal implications. 
